# A Deterministic Model for Q Fever Transmission Dynamics within Dairy Cattle Herds: Using Sensitivity Analysis and Optimal Controls

**DOI:** 10.1155/2020/6820608

**Published:** 2020-01-31

**Authors:** Joshua Kiddy K. Asamoah, Zhen Jin, Gui-Quan Sun, Michael Y. Li

**Affiliations:** ^1^Complex Systems Research Center, Shanxi University, Taiyuan 030006, China; ^2^Department of Mathematical and Statistical Sciences, University of Alberta, Edmonton, Alberta T6G 2G1, Canada

## Abstract

This paper presents a differential equation model which describes a possible transmission route for Q fever dynamics in cattle herds. The model seeks to ascertain epidemiological and theoretical inferences in understanding how to avert an outbreak of Q fever in dairy cattle herds (livestock). To prove the stability of the model's equilibria, we use a matrix-theoretic method and a Lyapunov function which establishes the local and global asymptotic behaviour of the model. We introduce time-dependent vaccination, environmental hygiene, and culling and then solve for optimal strategies. The optimal control strategies are necessary management practices that may increase animal health in a *Coxiella burnetii*-induced environment and may also reduce the transmission of the disease from livestock into the human population. The sensitivity analysis presents the relative importance of the various generic parameters in the model. We hope that the description of the results and the optimality trajectories provides some guidelines for animal owners and veterinary officers on how to effectively minimize the bacteria and control cost before/during an outbreak.

## 1. Introduction

Q fever is a bacterial disease which is caused by *Coxiella burnetii* (*C. burnetii*) [1, 2]. Q fever is a potential biological warfare agent being very infectious and very durable in the environment as well as capable of windborne spread [3]. *C. burnetii* affects a wide range of animals and also causes illness in humans [1, 2]. The bacteria are mostly found in farm animals such as goats, sheep, and cattle, but can also be found in cats, dogs, rodents, birds, and other wildlife [4, 5]. *Coxiella burnetii* in ruminants result in abortion, stillbirth, mastitis, infertility, premature delivery, and weak offspring [6–8]. Other clinical signs in animals may include, fever, mild coughing, anorexia, and rhinitis [9]. The bacteria are shed from an infected animal into the environment through urine, faeces, milk, and vaginal fluids, but most commonly, the bacterium is in the amniotic fluids and the placenta discharge during abortion or parturition of an infected animal [5]. Q fever is noted as the second most commonly reported laboratory infection with several recorded outbreaks involving 15 or more persons [3]. The disease dynamics in both animals and humans start primarily through inhalation of contaminated dust, contact with placenta discharge during an abortion, parturition of an infected animal, drinking unpasteurised infected milk, ingestion meat containing *Coxiella burnetii*, or contact with contaminated wool [3, 5, 10, 11]. Outbreaks typically occur following birth or abortion where the environment becomes contaminated with birthing fluids of an infected animal [3]. The bacteria contaminate dust and then are spread by the wind for long distances [3].

Q fever is a reemerging zoonosis in most parts of Europe, which has seen a sharp rise in recent years, especially in the Netherlands with a large number of human cases, attributed to livestock [12–14]. An outbreak of Q fever in the Netherlands between 2007 and 2010 resulted in about 4000 reported human cases, and efforts to end the epidemic resulted in the culling of more than 50,000 small ruminants and a temporary ban on breeding of animals [10]. A survey indicates that 82% of cows in some California dairies were seropositive, as well as 78% of coyotes, 55% of foxes, 53% of brush rabbits, and 22% of deer in Northern California [9]. Australia meat industry makes about $1 million losses annually as a result of Q fever [15]. Geographically, Q fever infection in animals has been detected in most countries, except, New Zealand, Iceland, French Polynesia, and Norway [3, 10].

In recent time, Q fever has received an increase in publicity on the potential source of transmission and possible preventative measures in both livestock and humans (see, for example, [11, 16] and the references therein). The research by De Rooij et al. [17] suggested that during any future epidemics, attention should be placed on a rapid source of identification, quantification of emission, accurate data collection, and smooth data exchange amongst relevant actors to enable effective risk assessment and risk management. Mori and Roest [18] pointed out that in achieving an effective control and management, there should be collective expertise from other scientific disciplines and stakeholder so that the expectations of farmers and the wider community can be met on the spread of the disease. Courcoul et al. [19] used the Bayesian approach to obtain key epidemiological parameters from field data and suggested that the values of the parameters obtained can be used to provide information for calibrating simulation models to assess control strategies for *C. burnetii*. Courcoul et al. [20] further proposed a model to assess the effectiveness of vaccination in dairy cattle and suggested that their model should be modified to simulate various control strategies such as environmental and hygienic measures.

In this sense, our goal is to use the parameter values presented in [19, 20] and some assumptions to study the impact of vaccination, environmental hygiene (cleaning/burying of placenta discharge after birth or abortion), and culling/isolation using ordinary differential equation model, sensitivity analysis, and optimal control theory. Thus, we systematically analysed the environmental transmission of the disease and then obtained mathematical and epidemiological properties, such as basic reproduction number, equilibrium points, local and global stability analysis, sensitivity analysis, and optimality conditions. The optimal control introduced is to obtain optimal trajectories that depict the most effective control measures and also account for the costs involved. Optimal control is a useful mathematical tool which has been recently used to determine optimal strategies for other infectious diseases, although not for Q fever.

The rest of the paper is arranged as follows: Section 2 presents a vaccination-induced model and mathematical analysis, which establishes the stability of the proposed model; in Section 3, we study the local and global sensitivity analysis of the model's parameters; in Section 4, we set up an optimal control problem; finally, in Section 5, we draw conclusions from this paper and assess what new insights this work gives to the body of knowledge on Q fever transmission dynamics and the way forward.

## 2. Basic Model for Indirect Transmission

The model is divided into susceptible *S*(*t*), asymptomatic (expose) *A*(*t*), vaccinated *V*(*t*), and symptomatic *I*(*t*) cattle, respectively, with an assumed bacteria- (*C. burnetii-*) induced environment *B*(*t*), where *t* represents time. The rate at which new cattle enter the susceptible population is denoted as Λ, *μ* is the constant rate of death, and thus 1/*μ* is the average lifetime. The asymptomatic cattle become symptomatic at a constant rate *α*, and so 1/*α* is the average asymptomatic period. The susceptible cattle get vaccinated at the rate *ν*, *θ* is the rate at which vaccinated cattle lose immunity and regain susceptibility. The symptomatic cattle are affected by an additional exit which may serve as a control measure at a constant rate *c* (culling/isolation rate). The rate of natural decay of the bacteria from the environment is denoted by *δ* and *ε* represents the rate of environmental hygiene, thus cleaning/burying of placenta discharge after birth or abortion. Asymptomatic cattle who develop temporal resistance to the bacteria become susceptible again at the rate *ρ*, if *ρ*=0 means no temporal resistance to the bacteria, and *ρ*=1 means 100% resistant to the bacteria (thus *I*=0), and hence throughout this paper, we assume 0 ≤ *ρ* < 1. We assume a mass action for the transmission of the disease, and thus susceptible cattle become asymptomatic at a proportional rate *βS*, where *β* is the effective rate of contracting the bacteria through indirect means (thus environmental transmission rate through inhaling contaminated dust/through grazing). The rate of inflow of the bacteria into the environment by both asymptomatic and symptomatic cattle is *η* (thus an assumed shedding rate). Here, the total cattle population for the vaccination-induced model is denoted as *N*=*S*+*V*+*A*+*I*. The following assumptions govern the vaccination-induced model:

- The parameters Λ, *β*, *η*, *α*, *ν*, *θ*, *ρ*, *μ*, *c*, *ε*, *δ* are all positive, and thus (Λ, *μ*, *η*, *α*, *δ*, *β*) > 0 and (*c*, *ρ*, *ε*, *θ*, *ν*) ≥ 0
- Newly purchased or new birth goes into the susceptible class only
- No permanent recovery
- A successfully vaccinated animal stays immune for a period of time

**Figure 1:**
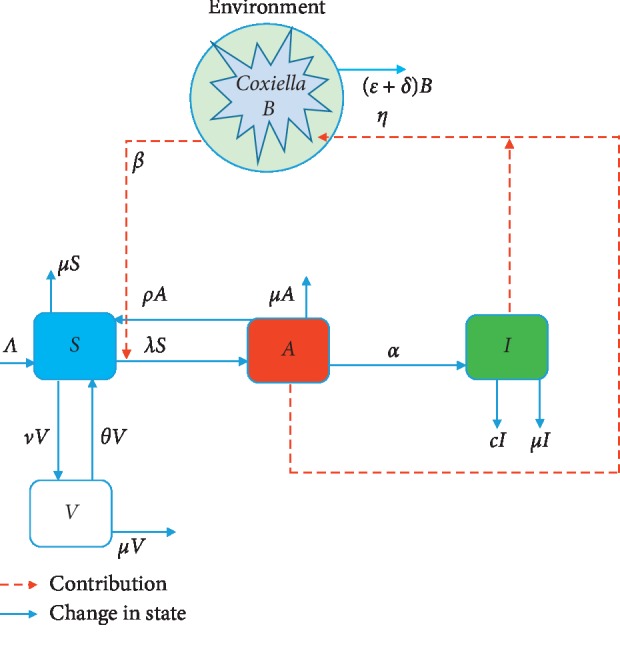
A flowchart of Q fever transmission dynamics within dairy cattle herds. The light blue box = susceptible cattle (*S*); red box = asymptomatic cattle (*A*); green box = symptomatic cattle (*I*); white box = vaccinated cattle (*V*); the presence of *Coxiella burnetii* in the environment (*B*) is encircled in blue.

Figure 1 depicts the compartmental diagrams of the disease in a community with control measures; model (1) gives the dynamical nature of the individual compartments with respect to time.

### 2.1. Basic Model and Analysis

(1) $$\begin{array}{c} \left\{\begin{array}{c} \frac{\text{d}S}{\text{d}t}=\Lambda -\beta BS-\left(\mu +\nu \right)S+\theta V+\rho A,\\ \frac{\text{d}V}{\text{d}t}=\nu S-\left(\theta +\mu \right)V,\\ \frac{\text{d}A}{\text{d}t}=\beta BS-\left(\mu +\alpha +\rho \right)A,\\ \frac{\text{d}I}{\text{d}t}=\alpha A-\left(c+\mu \right)I,\\ \frac{\text{d}B}{\text{d}t}=\eta \left(A+I\right)-\left(\varepsilon +\delta \right)B, \end{array}\right. \end{array}$$

with initial conditions

(2) $$\begin{array}{c} S\left(0\right)=S_{0}>0,\\ V\left(0\right)=V_{0}>0,\\ A\left(0\right)=A_{0}\geq 0,\\ I\left(0\right)=I_{0}\geq 0,\\ B\left(0\right)=B_{0}\geq 0, \end{array}$$

and total differential population

(3) $$\begin{array}{c} \left\{\begin{array}{c} \frac{\text{d}N}{\text{d}t}=\Lambda -\mu N-cI,\\ \frac{\text{d}B}{\text{d}t}=\eta \left(A+I\right)-\left(\varepsilon +\delta \right)B. \end{array}\right. \end{array}$$

### 2.2. Positivity and Feasible Region

**Lemma 1 .**

For any nonnegative initial data,

(4) $$\begin{array}{c} S\left(0\right)=S_{0}>0,\\ V\left(0\right)=V_{0}>0,\\ A\left(0\right)=A_{0}>0,\\ I\left(0\right)=I_{0}>0,\\ B\left(0\right)=B_{0}>0, \end{array}$$

model (1) has a unique global solution for all *t* ≥ 0.

**Lemma 2 .**

From the initial conditions and for epidemiological meaningfulness, we assume that all parameters are positive. Hence, the proposed model (1) will then be analysed in a feasible region given as

(5) $$\begin{array}{c} \Omega =\left\{\left(S,V,A,I,B\right)\in \left.{ℛ}_{+}^{5}\right|N\left(t\right)\leq \frac{\Lambda }{\mu },B\left(t\right)\leq \frac{\eta \Lambda }{\left(\varepsilon +\delta \right)\mu }\right\}. \end{array}$$

Now, we need to show that the region Ω is positively invariant, to support the assertion that the model is epidemiologically useful and that all its state variables are nonnegative as time increases and stays in Ω.

**Proof**

Let *X*=(*S*, *V*, *A*, *I*, *B*)^*𝒯*^ where *𝒯* denotes transposition, also if we replace *βB* with *M*, then model (1) can be written as (d*X*/d*t*)=*DX*+*Y*, where

(6) $$\begin{array}{c} D=\left(\begin{array}{ccccc} -\left(M+\mu +\nu \right) & \theta & \rho & 0 & 0\\ \nu & -\left(\theta +\mu \right) & 0 & 0 & 0\\ M & 0 & -\left(\mu +\alpha +\rho \right) & 0 & 0\\ 0 & 0 & \alpha & -\left(c+\mu \right) & 0\\ 0 & 0 & \eta & \eta & -\left(\varepsilon +\delta \right) \end{array}\right),\\ Y=\left(\begin{array}{c} \Lambda \\ 0\\ 0\\ 0\\ 0 \end{array}\right). \end{array}$$

Now, from the second equation in model (1), we have

(7) $$\begin{array}{c} \frac{\text{d}V}{\text{d}t}=\nu S-\left(\theta +\mu \right)V, \end{array}$$

applying the integration factor method and change of variables, we have

(8) $$\begin{array}{c} V\left(t\right)=V\left(0\right)e^{-\left(\theta +\mu \right)t}+e^{-\left(\theta +\mu \right)t}\nu \int_{0}^{t}S\left(p\right)e^{\left(\theta +\mu \right)p}\text{d}p\\ =e^{-\left(\theta +\mu \right)t}\left[V\left(0\right)+\nu \int_{0}^{t}S\left(p\right)e^{\left(\theta +\mu \right)p}\text{d}p\right]. \end{array}$$

From the third equation of model (1), we also obtain

(9) $$\begin{array}{c} A\left(t\right)=e^{-\left(\mu +\alpha +\rho \right)t}\left[A\left(0\right)+\beta \int_{0}^{t}B\left(p\right)S\left(p\right)e^{\left(\mu +\alpha +\rho \right)p}\text{d}p\right]. \end{array}$$

It follows from standard property that (d*V*/d*t*)|_*t*=*t*_0__ ≥ 0, (d*A*/d*t*)|_*t*=*t*_0__ ≥ 0, and (d*S*/d*t*)|_*t*=*t*_0__ ≥ 0, provided *S*(*t*_0_)=0, with similar properties being valid for *I*(*t*), *B*(*t*), which ensures that the state variable remains positive during the entire scope of the study (see [21] and Appendix A of [22]). Based on this, we see that matrix *D* has all its off-diagonal entries to be nonnegative and that matrix *Y* ≥ 0, which proves the properties of Metzler matrix [23]. Therefore, it implies that model (1) is positively invariant in *ℝ*_+_^5^. Finally, using equation (3), it can be shown that all solution sets of model (1) remain in the feasible region *Ω*, with the notation that 0 < *A*+*I* ≤ *N* and *S*+*A*+*I*+*V*=*N*. Hence, the proposed model is considered to be epidemiologically meaningful (mathematically well posed) and it is sufficient to say that all solutions of the Q fever model with initial conditions remain in the feasible region *Ω*.

### 2.3. Equilibrium Points and Basic Reproduction Number

The equilibrium points are the constant solutions to the differential equations in model (1), and these equilibrium points and the basic reproduction number play an important role in the long-term behaviour of the solution to the epidemiological model (1). The equilibrium points of model (1) are obtained by setting the right-hand side of model (1) to zero, which gives

(10) $$\begin{array}{c} \Lambda -\beta BS-\left(\mu +\nu \right)S+\theta V+\rho A=0, \end{array}$$

(11) $$\begin{array}{c} \nu S-\left(\theta +\mu \right)V=0, \end{array}$$

(12) $$\begin{array}{c} \beta BS-\left(\mu +\alpha +\rho \right)A=0, \end{array}$$

(13) $$\begin{array}{c} \alpha A-\left(c+\mu \right)I=0, \end{array}$$

(14) $$\begin{array}{c} \eta \left(A+I\right)-\left(\varepsilon +\delta \right)B=0. \end{array}$$

The two main equilibria considered here are the disease-free equilibrium and endemic equilibrium. We obtained the disease-free equilibrium by assuming that the disease-induced states *A*=*I*=0 and a free *C. burnetii* environment and thus *B*=0. Therefore, by solving for the nonzero state variables in equations (10) and (11), a unique disease-free equilibrium is obtained as follows:

(15) $$\begin{array}{c} E_{0}≔\left(S^{0},V^{0},A^{0},I^{0},B^{0}\right)=\left(\frac{\Lambda \left(\mu +\theta \right)}{\mu \left(\mu +\nu +\theta \right)},\frac{\Lambda \nu }{\mu \left(\mu +\nu +\theta \right)},0,0,0\right). \end{array}$$

#### 2.3.1. Basic Reproduction Numbers *ℛ*_*C*_ and *ℛ*_0_

**Figure 2:**
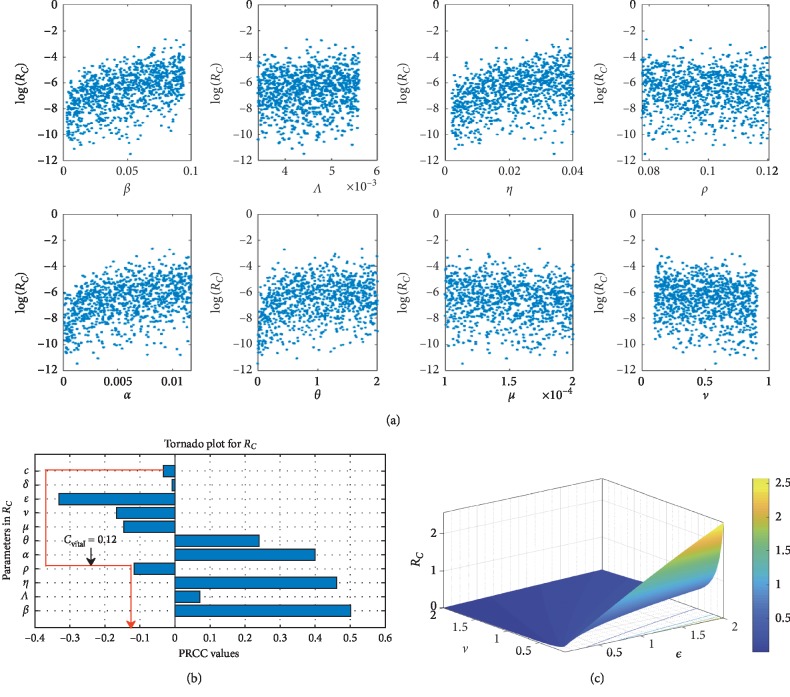
(a) The scatter diagrams for some selected parameters in *ℛ*_*C*_ (scatter plots of *β*, Λ, *η*, *ρ*, *α*, *θ*, *μ*, and *ν* in *ℛ*_*C*_). (b) Perfect rank relations of the parameters in *ℛ*_*C*_ (Tornado plot of parameters in *ℛ*_*C*_), (c) The coupling effect of both vaccination and environmental hygiene on *ℛ*_*C*_ (3D plot of *ε* and *vℛ*_*C*_).

The basic reproduction number *ℛ*_0_ is obtained by using the disease-free equilibrium points in terms of the original parameters of the model; epidemiologically, the reproduction number informs us on the number of secondary infections produced when a single infectious individual is introduced into a completely susceptible population. Here, we define the basic reproduction number as the number of secondary infections produced in a completely susceptible cattle population when the organism is released into the environment. Also, the analytic nature of the basic reproduction number gives clues on parameters in the model that contributes to the spread of the disease. Hence, with the aid of the next-generation matrix *FV*^−1^ as introduced by [24, 25] and the disease-free equilibrium (15), the epidemiological reproduction numbers for model (1) are as follows:

(16) $$\begin{array}{c} {ℛ}_{C}=\frac{\beta \eta \Lambda \left(\theta +\mu \right)\left(\alpha +c+\mu \right)}{\mu \left(\mu +\alpha +\rho \right)\left(\theta +\mu +\nu \right)\left(\varepsilon +\delta \right)\left(c+\mu \right)}, \end{array}$$

(17) $$\begin{array}{c} {ℛ}_{0}=\frac{\beta \eta \Lambda \left(\alpha +\mu \right)}{\mu^{2}\left(\mu +\alpha +\rho \right)\delta }, \end{array}$$

where equation (16) is called the control reproduction number *ℛ*_*C*_ and equation (17) is the basic reproduction number without controls *ℛ*_0_ (thus *c*=0,  *θ*=0, *ν*=0, *ε*=0). From the basic reproduction numbers, it is evidential that there exists a linear relationship between the rate of environmental transmission *β* and shedding rate *η*, and hence, this shows that an increase in bacterial load in the environment may have a corresponding increase in the transmission process. This assertion has been numerically varied using Figure 2(b) in Section 3.2.1.

#### 2.3.2. Endemic Equilibrium Point

Now, taking *S*, *V*, *A*, *I*, *B* > 0 in the feasible set *Ω*, and using equations (10)–(14) together with (16), we obtain the following endemic equilibrium point *E*^*∗*^≔(*S*^*∗*^, *V*^*∗*^, *A*^*∗*^, *I*^*∗*^, *B*^*∗*^). Thus

(18) $$\begin{array}{c} S^{*}=\frac{\left(\mu +\alpha +\rho \right)\left(\varepsilon +\delta \right)\left(c+\mu \right)}{\beta \eta \left(\alpha +c+\mu \right)},\\ V^{*}=\frac{S^{*}\nu }{\left(\theta +\mu \right)},\\ A^{*}=\frac{\left({ℛ}_{C}-1\right)}{\beta \eta \left(\theta +\mu \right)\left(\mu +\alpha \right)\left(\alpha +c+\mu \right)},\\ I^{*}=\frac{\alpha \left({ℛ}_{C}-1\right)}{\beta \eta \left(c+\mu \right)\left(\theta +\mu \right)\left(\mu +\alpha \right)\left(\alpha +c+\mu \right)},\\ B^{*}=\frac{\left({ℛ}_{C}-1\right)}{\beta \left(\mu +c\right)\left(\varepsilon +\delta \right)\left(\theta +\mu \right)\left(\mu +\alpha \right)}. \end{array}$$

The endemic equilibrium has a unique equilibrium point if the vaccination-induced reproduction number is greater than unity (*ℛ*_*C*_ > 1). In the next subsection, we investigate the stability of the equilibrium points.

### 2.4. Stability Analysis

In this section, we focus on establishing the local and global stability of the model equilibria. To obtain the local stability of the disease-free equilibrium, we constructed a linearized Jacobian matrix *J*_1_, evaluated at the disease-free equilibrium, which gives

(19) $$\begin{array}{c} J_{1}=\left(\begin{array}{ccc} -\left(\mu +\alpha +\rho \right) & 0 & \frac{\beta \Lambda \left(\theta +\mu \right)}{\mu \left(\theta +\mu +\nu \right)}\\ \alpha & -\left(c+\mu \right) & 0\\ \eta & \eta & -\left(\varepsilon +\delta \right) \end{array}\right), \end{array}$$

where *J*_1_=*F* − *V*, *J*_1_ is irreducible and nonnegative. The simple eigenvalue of *J*_1_ is denoted as *s*(*J*_1_) which is obtained from the relation *s*(*J*_1_)=max{Re*λ* : Re*λ* is the real part of eigenvalue of matrix *J*_1_}. The following equivalence holds:

(20) $$\begin{array}{c} {ℛ}_{C}>1⟺s\left(J_{1}\right)>0,\\ {ℛ}_{C}<1⟺s\left(J_{1}\right)<0,\;\left(\text{see},\text{ Theorem }2\text{ of }\left[24\right]\text{Section }3\right). \end{array}$$

Using Theorem 2 of van den Driessche and Watmough [24], we see that assumptions (*A*1) − (*A*4) are satisfied, and also (*A*5) is satisfied if all the eigenvalues of matrix *J*|_*E*_0__ have negative real parts, and thus

(21) $$\begin{array}{c} {\left.J\right|}_{E_{0}}=\left(\begin{array}{cc} J_{1} & 0\\ J_{3} & J_{4} \end{array}\right), \end{array}$$

where *J*_4_=−*F*, and $J_{4}=\left(\begin{array}{cc} -\left(\mu +\nu \right) & \theta \\ \nu & \left(\theta +\mu \right) \end{array}\right)$.

The simple eigenvalue of *J*_4_ is *s*(*J*_4_)=max{−(*μ*+*ν*+*θ*), −*μ*}. Hence, model (1) fully satisfies the assumptions (*A*(1) − *A*(5)) of van den Driessche and Watmough [24], and therefore, the disease-free equilibrium of the Q fever model is locally asymptotically stable if *ℛ*_*C*_ < 1 and unstable if *ℛ*_*C*_ > 1. The below theorem addresses the global stability of the disease-free equilibrium.

**Theorem 1 .**

If *ℛ*_*C*_ ≤ 1, then the disease-free equilibrium of model (1) is globally asymptotically stable.

**Proof**

Applying Theorem 2.1 of the matrix-theoretic method in [26], the global stability of the disease-free equilibrium (*E*_0_) can be established by constructing a Lyapunov function Φ=*w*^*T*^*V*^−1^*x*, since *V*^−1^*F* is reducible (the third column is the only nonzero column), *w*^*T*^ is the left Perron eigenvector of the matrix *V*^−1^*F*, and *x*=(*A*, *I*, *B*)^*T*^ is the disease-induced classes. Algebraic operation of *V*^−1^*F* gives

(22) $$\begin{array}{c} V^{-1}F=\left(\begin{array}{ccc} 0 & 0 & \frac{\beta S^{0}}{\left(\mu +\alpha +\rho \right)}\\ 0 & 0 & \frac{\alpha \beta S^{0}}{\left(\mu +\alpha +\rho \right)\left(c+\mu \right)}\\ 0 & 0 & \frac{\beta \eta S^{0}\left(\alpha +c+\mu \right)}{\left(\mu +\alpha +\rho \right)\left(c+\mu \right)\left(\varepsilon +\delta \right)} \end{array}\right), \end{array}$$

where *S*^0^=Λ(*μ*+*θ*)/*μ*(*μ*+*ν*+*θ*). Therefore, by Theorem 5.1 of [26], the left Perron eigenvector *w*^*T*^ of *V*^−1^*F* for the largest eigenvalue is defined as

(23) $$\begin{array}{c} \left(0,0,w_{3}\right)V^{-1}F={ℛ}_{C}\left(0,0,w_{3}\right). \end{array}$$

This gives

(24) $$\begin{array}{c} {ℛ}_{C}w_{3}=w_{3}\frac{\beta \eta S^{0}\left(\alpha +c+\mu \right)}{\left(\mu +\alpha +\rho \right)\left(c+\mu \right)\left(\varepsilon +\delta \right)}, \end{array}$$

which implies that *w*_3_=1; therefore, *w*^*T*^=(0,0,1). Now simplifying Φ=*w*^*T*^*V*^−1^*x* yields

(25) $$\begin{array}{c} \Phi =\frac{\eta \left(\alpha +c+\mu \right)A+\eta \left(\mu +\alpha +\rho \right)I+\left(\mu +\alpha +\rho \right)\left(c+\mu \right)B}{\left(\mu +\alpha +\rho \right)\left(c+\mu \right)\left(\varepsilon +\delta \right)},\\ =\frac{{ℛ}_{C}}{\beta S^{0}}\left[A+\left(\frac{\left(\mu +\alpha +\rho \right)}{\left(\alpha +c+\mu \right)}\right)I+\frac{\left(\mu +\alpha +\rho \right)\left(c+\mu \right)}{\eta \left(\alpha +c+\mu \right)}B\right]. \end{array}$$

Using [26], the derivative of Φ along the solution of model (1) is illustrated as follows:

(26) $$\begin{array}{c} \frac{\text{d}\Phi }{\text{d}t}=\left({ℛ}_{C}-1\right)w^{T}x-w^{T}V^{-1}f\left(x,S\right), \end{array}$$

where *f*(*x*, *S*)≔(*F* − *V*)*x* − *ℱ*(*x*, *S*)+*𝒱*(*x*, *S*). Therefore, after some algebra, we have

(27) $$\begin{array}{c} f\left(x,S\right)=\left(\begin{array}{c} \beta B\left(S^{0}-S\right)\\ 0\\ 0 \end{array}\right),\;\text{for }S^{0}\geq S. \end{array}$$

Now using equation (26) and some algebraic simplification leads to

(28) $$\begin{array}{c} \frac{\text{d}\Phi }{\text{d}t}=\left({ℛ}_{C}-1\right)B-\frac{{ℛ}_{C}}{S^{0}}B\left(S^{0}-S\right). \end{array}$$

Therefore, (dΦ/d*t*) ≤ 0 provided *ℛ*_*C*_ ≤ 1 and *S*^0^ ≥ *S*. Furthermore, (dΦ/d*t*) ≤ 0 implies that *ℛ*_*C*_=1 and *B*=0 or *S*=*S*^0^. It can be shown that the only invariant set where *B*=0 or *S*=*S*^0^ is a singleton of the set {*S*^0^, 0,0,0}. Since the four state variables used in constructing the Lyapunov functional lead to global stability, it then means that *V*(*t*)⟶*V*^0^ as *t*⟶*∞*. Hence, by [27] (LaSalle's invariance principle), the disease-free equilibrium for the vaccination model is globally asymptotically stable. The ramification of this global stability in the presence of vaccination implies that no matter how many initial asymptomatic and symptomatic cattle in the population, the disease cannot be established (persist) and eventually dies out. It also indicates that no matter how many initial bacterial loads in the environment, the disease will die out from the population in the long round.

#### 2.4.1. Global Stability of Endemic Equilibrium

The nature of the endemic equilibrium *E*^*∗*^ indicates that for *ℛ*_*C*_ > 1, then there exists a unique endemic equilibrium resulting in an unstable disease-free equilibrium. To establish the global stability of *E*^*∗*^, here we explore a special case (simpler case) of the model (1), where *ρ*=0. Let *Ω*_0_={(*S*, *V*, *A*, *I*) ∈ *Ω* : *A*=*I*=0} be the stable manifold of *E*_0_. Therefore, we claim the following result.

**Theorem 2 .**

The unique endemic equilibrium of the model (1) is globally asymptotically stable in *Ω*/*Ω*_0_ whenever *ℛ*_*C*_ > 1 and *ρ*=0.

**Proof**

At the endemic equilibrium state of model (1) with *ρ*=0, the following equalities

(29) $$\begin{array}{c} \Lambda -\beta B^{*}S^{*}-\left(\mu +\nu \right)S^{*}+\theta V^{*}=0, \end{array}$$

(30) $$\begin{array}{c} \nu S^{*}-\left(\theta +\mu \right)V^{*}=0, \end{array}$$

(31) $$\begin{array}{c} \beta B^{*}S^{*}-\left(\mu +\alpha \right)A^{*}=0, \end{array}$$

(32) $$\begin{array}{c} \alpha A^{*}-\left(c+\mu \right)I^{*}=0, \end{array}$$

(33) $$\begin{array}{c} \eta \left(A^{*}+I^{*}\right)-\left(\varepsilon +\delta \right)B^{*}=0, \end{array}$$

hold, which further leads to

(34) $$\begin{array}{c} \left\{\begin{array}{c} \left(\mu +\nu \right)=\frac{\Lambda -\beta B^{*}S^{*}+\theta V^{*}}{S^{*}},\\ \left(\theta +\mu \right)=\frac{\nu S^{*}}{V^{*}},\\ \left(\mu +\alpha \right)=\frac{\beta B^{*}S^{*}}{A^{*}},\\ \left(c+\mu \right)=\frac{\alpha A^{*}}{I^{*}},\\ \left(\varepsilon +\delta \right)=\frac{\eta \left(A^{*}+I^{*}\right)}{B^{*}}. \end{array}\right. \end{array}$$

Substituting equation (34) into model (1) result in model (35), in which its stability is the same as model (1):

(35) $$\begin{array}{c} \left\{\begin{array}{c} \frac{\text{d}S}{\text{d}t}=S\left[\frac{\Lambda }{S^{*}}\left(\frac{S^{*}}{S}-1\right)+\frac{\theta V^{*}}{S^{*}}\left(\frac{VS^{*}}{V^{*}S}-1\right)-\beta B^{*}\left(\frac{B}{B^{*}}-1\right)\right],\\ \frac{\text{d}V}{\text{d}t}=\frac{\nu S^{*}V}{V^{*}}\left(\frac{SV^{*}}{VS^{*}}-1\right),\\ \frac{\text{d}A}{\text{d}t}=\frac{\beta B^{*}S^{*}A}{A^{*}}\left(\frac{BA^{*}S}{B^{*}S^{*}A}-1\right),\\ \frac{\text{d}I}{\text{d}t}=\frac{\alpha A^{*}I}{I^{*}}\left(\frac{AI^{*}}{IA^{*}}-1\right),\\ \frac{\text{d}B}{\text{d}t}=\frac{\eta B}{B^{*}}\left[A^{*}\left(\frac{B^{*}A}{A^{*}B}-1\right)+I^{*}\left(\frac{IB^{*}}{I^{*}B}-1\right)\right]. \end{array}\right. \end{array}$$

Now, we define a Lyapunov function as

(36) $$\begin{array}{c} L_{1}=\left(S-S^{*}+S^{*}\ln\frac{S}{S^{*}}\right)+\left(V-V^{*}+V^{*}\ln\frac{V}{V^{*}}\right)+\left(A-A^{*}+A^{*}\ln\frac{A}{A^{*}}\right)\\ +k_{1}\left(I-I^{*}+I^{*}\ln\frac{I}{I^{*}}\right)+k_{2}\left(B-B^{*}+B^{*}\ln\frac{B}{B^{*}}\right), \end{array}$$

where *k*_1_ and *k*_2_ are positive constants to be determined, and thus *k*_1_ > 0,  *k*_2_ > 0. The time derivative of the Lyapunov function along with the solution of model (35) yields the following equation:

(37) $$\begin{array}{c} \frac{\text{d}L_{1}}{\text{d}t}=\left(1-\frac{S^{*}}{S}\right)S\left[\frac{\Lambda }{S^{*}}\left(\frac{S^{*}}{S}-1\right)+\frac{\theta V^{*}}{S^{*}}\left(\frac{VS^{*}}{V^{*}S}-1\right)-\beta B^{*}\left(\frac{B}{B^{*}}-1\right)\right]\\ +\left(1-\frac{V^{*}}{V}\right)\frac{\nu S^{*}V}{V^{*}}\left(\frac{SV^{*}}{VS^{*}}-1\right)+\left(1-\frac{A^{*}}{A}\right)\frac{\beta B^{*}S^{*}A}{A^{*}}\left(\frac{BA^{*}S}{B^{*}S^{*}A}-1\right)\\ +k_{1}\left(1-\frac{I^{*}}{I}\right)\frac{\alpha A^{*}I}{I^{*}}\left(\frac{AI^{*}}{IA^{*}}-1\right)+k_{2}\left(1-\frac{B^{*}}{B}\right)\frac{\eta B}{B^{*}}\left[A^{*}\left(\frac{B^{*}A}{A^{*}B}-1\right)+I^{*}\left(\frac{IB^{*}}{I^{*}B}-1\right)\right]. \end{array}$$

Simplifying the above expression and rearranging lead to

(38) $$\begin{array}{c} \frac{\text{d}L_{1}}{\text{d}t}=\Lambda \left(S-S^{*}\right)\left(\frac{1}{S}-\frac{1}{S^{*}}\right)+\theta \left(S-S^{*}\right)\left(\frac{V}{S}-\frac{V^{*}}{S^{*}}\right)+\nu \left(V-V^{*}\right)\left(\frac{S}{V}-\frac{S^{*}}{V^{*}}\right)\\ -\left(S-S^{*}\right)\left[\beta B^{*}\left(\frac{B}{B^{*}}-1\right)\right]+\left(A-A^{*}\right)\frac{\beta B^{*}S^{*}}{A^{*}}\left(\frac{BA^{*}S}{B^{*}S^{*}A}-1\right)\\ +k_{1}\left(I-I^{*}\right)\frac{\alpha A^{*}}{I^{*}}\left(\frac{AI^{*}}{IA^{*}}-1\right)+k_{2}\left(B-B^{*}\right)\frac{\eta }{B^{*}}\left[A^{*}\left(\frac{B^{*}A}{A^{*}B}-1\right)+I^{*}\left(\frac{IB^{*}}{I^{*}B}-1\right)\right]. \end{array}$$

We now let

(39) $$\begin{array}{c} L_{1}=\Lambda \left(S-S^{*}\right)\left(\frac{1}{S}-\frac{1}{S^{*}}\right)+\theta \left(S-S^{*}\right)\left(\frac{V}{S}-\frac{V^{*}}{S^{*}}\right)+\nu \left(V-V^{*}\right)\left(\frac{S}{V}-\frac{S^{*}}{V^{*}}\right), \end{array}$$

(40) $$\begin{array}{c} L_{2}=-\left(S-S^{*}\right)\left[\beta B^{*}\left(\frac{B}{B^{*}}-1\right)\right]+\left(A-A^{*}\right)\frac{\beta B^{*}S^{*}}{A^{*}}\left(\frac{BA^{*}S}{B^{*}S^{*}A}-1\right) \end{array}$$

Making Λ and *ν* the subject in equations (29) and (30), respectively, and substituting into (39) with series of simplification give

(41) $$\begin{array}{c} L_{1}=\mu S^{*}\left(2-\frac{S}{S^{*}}-\frac{S^{*}}{S}\right)+\mu V^{*}\left(3-\frac{S^{*}}{S}-\frac{SV^{*}}{VS^{*}}-\frac{V}{V^{*}}\right)+\theta V^{*}\left(2-\frac{VS^{*}}{SV^{*}}-\frac{V^{*}S}{S^{*}V}\right). \end{array}$$

Further elaboration on *ℒ*_2_ results in

(42) $$\begin{array}{c} L_{2}=k_{2}\eta \left[A^{*}\left(1+\frac{A}{A^{*}}-\frac{B}{B^{*}}-\frac{AB^{*}}{BA^{*}}\right)+I^{*}\left(1+\frac{I}{I^{*}}-\frac{B}{B^{*}}-\frac{B^{*}I}{BI^{*}}\right)\right]\\ +\alpha k_{1}A^{*}\left[\frac{A}{A^{*}}-\frac{I}{I^{*}}-\frac{AI^{*}}{IA^{*}}+1\right]+\beta B^{*}S^{*}\left(\frac{B}{B^{*}}-\frac{A}{A^{*}}+\frac{S}{S^{*}}-\frac{BSA^{*}}{B^{*}S^{*}A}\right). \end{array}$$

To obtain *k*_1_, *k*_2_, we make the following assertion by equating the constant coefficients of the terms *A*/*A*^*∗*^, *I*/*I*^*∗*^ and *B*/*B*^*∗*^ in *ℒ*_2_ to zero, thus

(43) $$\begin{array}{c} k_{2}\eta A^{*}+\alpha k_{1}A^{*}-\beta B^{*}S^{*}=0, \end{array}$$

(44) $$\begin{array}{c} k_{2}\eta I^{*}-\alpha k_{1}A^{*}=0, \end{array}$$

(45) $$\begin{array}{c} \beta B^{*}S^{*}-k_{2}\eta \left(A^{*}+I^{*}\right)=0. \end{array}$$

Solving (44) and (45), we obtain *k*_1_=*βB*^*∗*^*S*^*∗*^*I*^*∗*^/*αA*^*∗*^(*A*^*∗*^+*I*^*∗*^) and *k*_2_=*βB*^*∗*^*S*^*∗*^/*η*(*A*^*∗*^+*I*^*∗*^). Replacing *k*_1_, *k*_2_ into (42) and simplifying yield

(46) $$\begin{array}{c} L_{2}=\frac{\beta B^{*}S^{*}I^{*}}{\left(A^{*}+I^{*}\right)}\left(2+\frac{A}{A^{*}}-\frac{B}{B^{*}}-\frac{B^{*}I}{BI^{*}}-\frac{AI^{*}}{A^{*}I}\right)+\frac{\beta B^{*}S^{*}A^{*}}{\left(A^{*}+I^{*}\right)}\left(1+\frac{A}{A^{*}}-\frac{B}{B^{*}}-\frac{B^{*}A}{BA^{*}}\right)\\ +\beta BS^{*}\left(1+\frac{B^{*}S}{BS^{*}}-\frac{B^{*}A}{A^{*}B}-\frac{SA^{*}}{S^{*}A}\right). \end{array}$$

Hence, adding equations (41) and (46), we have

(47) $$\begin{array}{c} \frac{\text{d}L_{1}}{\text{d}t}=\mu S^{*}\left(2-\frac{S}{S^{*}}-\frac{S^{*}}{S}\right)+\mu V^{*}\left(3-\frac{S^{*}}{S}-\frac{SV^{*}}{VS^{*}}-\frac{V}{V^{*}}\right)+\theta V^{*}\left(2-\frac{VS^{*}}{SV^{*}}-\frac{V^{*}S}{S^{*}V}\right)\\ \frac{\beta B^{*}S^{*}I^{*}}{\left(A^{*}+I^{*}\right)}\left(2+\frac{A}{A^{*}}-\frac{B}{B^{*}}-\frac{B^{*}I}{BI^{*}}-\frac{AI^{*}}{A^{*}I}\right)+\frac{\beta B^{*}S^{*}A^{*}}{\left(A^{*}+I^{*}\right)}\left(1+\frac{A}{A^{*}}-\frac{B}{B^{*}}-\frac{B^{*}A}{BA^{*}}\right)\\ +\beta BS^{*}\left(1+\frac{B^{*}S}{BS^{*}}-\frac{B^{*}A}{A^{*}B}-\frac{SA^{*}}{S^{*}A}\right), \end{array}$$

(48) $$\begin{array}{c} \frac{\text{d}L_{1}}{\text{d}t}=\mu S^{*}\left(2-\frac{S}{S^{*}}-\frac{S^{*}}{S}\right)+\mu V^{*}\left(3-\frac{S^{*}}{S}-\frac{SV^{*}}{VS^{*}}-\frac{V}{V^{*}}\right)+\theta V^{*}\left(2-\frac{VS^{*}}{SV^{*}}-\frac{V^{*}S}{S^{*}V}\right)\\ \frac{\beta B^{*}S^{*}I^{*}}{\left(A^{*}+I^{*}\right)}D_{0}+\frac{\beta B^{*}S^{*}A^{*}}{\left(A^{*}+I^{*}\right)}D_{1}+\beta BS^{*}D_{2}, \end{array}$$

where

(49) $$\begin{array}{c} D_{0}=\left(2+\frac{A}{A^{*}}-\frac{B}{B^{*}}-\frac{B^{*}I}{BI^{*}}-\frac{AI^{*}}{A^{*}I}\right),\\ D_{1}=\left(1+\frac{A}{A^{*}}-\frac{B}{B^{*}}-\frac{B^{*}A}{BA^{*}}\right),\\ D_{2}=\left(1+\frac{B^{*}S}{BS^{*}}-\frac{B^{*}A}{A^{*}B}-\frac{SA^{*}}{S^{*}A}\right). \end{array}$$

Thus, if *D*_0_ ≤ 0,  *D*_1_ ≤ 0,  *D*_2_ ≤ 0, and from the fact that the arithmetic mean is greater than or equal to the geometric mean, then it follows that (d*L*_1_/d*t*) ≤ 0, provided the coefficients *μS*^*∗*^,  *μV*^*∗*^,  *θV*^*∗*^,  *βB*^*∗*^*S*^*∗*^*I*^*∗*^/(*A*^*∗*^+*I*^*∗*^),  *βB*^*∗*^*S*^*∗*^*A*^*∗*^/(*A*^*∗*^+*I*^*∗*^),  *βBS*^*∗*^ are nonnegative. With the claim that

(50) $$\begin{array}{c} w\left(x\right)=x-1-\text{ln}\left(x\right), \end{array}$$

it leads to

(51) $$\begin{array}{c} D_{0}=w\left(\frac{A}{A^{*}}\right)-w\left(\frac{B}{B^{*}}\right)-w\left(\frac{B^{*}I}{BI^{*}}\right)-w\left(\frac{AI^{*}}{A^{*}I}\right),\\ D_{1}=w\left(\frac{A}{A^{*}}\right)-w\left(\frac{B}{B^{*}}\right)-w\left(\frac{B^{*}A}{BA^{*}}\right),\\ D_{2}=w\left(\frac{B^{*}S}{BS^{*}}\right)-w\left(\frac{B^{*}A}{A^{*}B}\right)-w\left(\frac{SA^{*}}{S^{*}A}\right). \end{array}$$

To verify the above expression, the definition of *w*(*x*) and properties of logarithms were used to simplify the expression on the right hand of *D*_*i*_,  *i*=0,1,2. Then applying the proposition *A*1 of [28] gives

(52) $$\begin{array}{c} D_{0}\leq -w\left(\frac{B}{B^{*}}\right)-w\left(\frac{B^{*}I}{BI^{*}}\right)-w\left(\frac{AI^{*}}{A^{*}I}\right)\leq 0,\\ D_{1}\leq -w\left(\frac{B}{B^{*}}\right)-w\left(\frac{B^{*}A}{BA^{*}}\right)\leq 0,\\ D_{2}\leq -w\left(\frac{B^{*}A}{A^{*}B}\right)-w\left(\frac{SA^{*}}{S^{*}A}\right)\leq 0. \end{array}$$

It is evidential that (d*L*_1_(*S*, *V*, *A*, *I*, *B*)/d*t*)=0 only holds when *S*=*S*^*∗*^,  *V*=*V*^*∗*^,  *A*=*A*^*∗*^,  *I*=*I*^*∗*^,  *B*=*B*^*∗*^ and that *E*^*∗*^ is the only equilibrium state of model (1) on its line. Therefore, by Lyapunov–LaSalle asymptotic stability theorem [27], the positive equilibrium state *E*^*∗*^ is globally asymptotically stable in the positive region *ℝ*_+_^5^ whenever *ℛ*_*C*_ > 1 and *ρ*=0.

## 3. Sensitivity Analysis

Here, we apply the concept of sensitivity analysis to ascertain the relative importance of each generic parameter in the basic reproduction number and the disease-induced state variables, *E*^*∗*^. Secondly, with parameter values from published papers and some realistic assumptions, we obtain both analytic and numerical values of the various parameters in *ℛ*_*C*_. The analytic expressions obtained can be used to shed some light on how to control the onset of the disease in variant localities, if and only if the dynamics follow model (1). Parameter values were obtained from [19, 20], which were then converted into days; this is to enable us to see the daily trajectories of the model. The estimated parameters were calculated based on the life expectancy of dairy cattle and the natural decay rate of the bacteria from dust/soil (which ranges from 120 to 150 days [9]), and finally, we used the gestation period of cattle to denote the rate of new inflow of susceptible cattle/recruited cattle. All parameters used are given in Tables 1 and 2, respectively.

### 3.1. The Impact of Transition Rate *ρ* on the Reproduction Number

Taking the partial derivative of equation (17) with respect to *ρ* gives the relative importance of the discontinuation rate *ρ* (the feedback term) and thus the rate at which asymptomatic cattle build antibiotics and reenter the susceptible class:

(53) $$\begin{array}{c} \frac{\partial {ℛ}_{0}}{\partial \rho }=-\frac{\Lambda \left(\alpha +\mu \right)\beta \eta }{{\left(\alpha +\mu +\rho \right)}^{2}\delta \mu^{2}}<0. \end{array}$$

Hence, the basic reproduction number *ℛ*_0_ without culling, vaccination, and environmental hygiene is a decreasing factor of the parameter *ρ* as shown in Subsection 2.4.1. This implies that the basic reproduction number decreases with increase in the value of *ρ*. Consequently, taking the limit of *ℛ*_0_ as *ρ* becomes large gives

(54) $$\begin{array}{c} \underset{\rho ⟶\infty }{\text{lim}}{ℛ}_{0}=0. \end{array}$$

This means that any attempt to increase *ρ* will result in reducing the number of secondary infections, thereby eradicating the disease in the absence of other control measures, but a low rate of *ρ* will produce a high rate of asymptomatic cattle which may require effective control measures as shown in Section 4. From this, we infer that the development of any daily antibody boosters for dairy cattle (livestock) will help curtail the reproduction losses caused by *C. burnetii* and other bacterial diseases.

### 3.2. Calculation of Sensitivity/Elasticity Indices

Fundamentally, the onset of disease transmission is directly associated with the basic/control reproduction number *ℛ*_*C*_, and the prevalence of the disease is associated with the endemic equilibrium point *E*^*∗*^ [29]. The sensitivity indices for the state variables are calculated as follows:

(55) $$\begin{array}{c} {ϒ}_{p_{i}}^{X_{i}}=\frac{\partial X_{i}}{\partial p_{i}}\times \frac{p_{i}}{X_{i}}, \end{array}$$

where *X*_*i*_ represent the equilibrium points at the endemic state and *p*_*i*_ are the corresponding parameters in *X*_*i*_ [30]. For simplicity, the numerical values of the endemic state variables are shown in Table 2 without analytic expression due to the complex nature of the analytic result. Similarly, the sensitivity/elasticity indices of *ℛ*_*C*_ to the parameters in the model are also defined as follows:

(56) $$\begin{array}{c} {ϒ}_{p_{i}}^{{ℛ}_{C}}=\frac{\partial {ℛ}_{C}}{\partial p_{i}}\times \frac{p_{i}}{{ℛ}_{C}}, \end{array}$$

where the meaning of *ℛ*_*C*_ and *p*_*i*_ remains unchanged. Therefore

(57) $$\begin{array}{c} {ϒ}_{\beta }^{{ℛ}_{C}}=1,\\ {ϒ}_{\Lambda }^{{ℛ}_{C}}=1,\\ {ϒ}_{\eta }^{{ℛ}_{C}}=1,\\ {ϒ}_{\rho }^{{ℛ}_{C}}=-\frac{\rho }{\alpha +\mu +\rho },\\ {ϒ}_{\alpha }^{{ℛ}_{C}}=-\frac{\alpha \left(c-\rho \right)}{\left(\alpha +c+\mu \right)\left(\alpha +\mu +\rho \right)},\\ {ϒ}_{\nu }^{{ℛ}_{C}}=-\frac{\nu }{\mu +\nu +\theta },\\ {ϒ}_{\theta }^{{ℛ}_{C}}=\frac{\nu \theta }{\left(\mu +\theta \right)\left(\mu +\nu +\theta \right)},\\ {ϒ}_{\varepsilon }^{{ℛ}_{C}}=-\frac{\varepsilon }{\delta +\varepsilon },\\ {ϒ}_{\delta }^{{ℛ}_{C}}=-\frac{\delta }{\delta +\varepsilon },\\ {ϒ}_{c}^{{ℛ}_{C}}=-\frac{\alpha c}{\left(c+\mu \right)\left(\alpha +c+\mu \right)},\\ {ϒ}_{\mu }^{{ℛ}_{C}}=-\mu \left[\frac{1}{\alpha +\mu +\rho }+\frac{1}{\mu +\nu +\theta }+\frac{1}{c+\mu }-\frac{1}{\mu +\theta }+\frac{1}{\mu }-\frac{1}{\alpha +c+\mu }\right]. \end{array}$$

**Remark 1 .**

*ϒ*_*α*_^*ℛ*_*C*_^ < 0 whenever *c* > *ρ*, this implies that an increase in culling/isolation of symptomatic cattle from farms during an outbreak will reduce the impact of *α*, thereby reducing the number of secondary infections and bacterial load in the environment as shown in Table 2. This further suggests that in choosing between culling and antibiotic booster during an outbreak, it is prudent to focus on culling/isolation.

In Table 2, we obtain numerical values which indicate the relative importance of parameters in *ℛ*_*C*_ and the state variables at the endemic stage when other parameters are kept constant (depicting the local sensitivity of parameters in model (1)). The result reveals that *β*, *λ*, *η* and *θ* influence the stability of the disease and are also closely related. Thus, a 1% increase in *β*, *λ*, *η*, and *θ* will have a corresponding percentage increase in the basic reproduction number. Among the control measures implemented, the elasticity index in Table 2 indicates that the most efficient method in the infected classes will be vaccination *ν* and the most efficient method of reducing the bacteria from the environment may be a good environmental hygiene *ε*. The total efficacy of these control measures will be shown in Section 4. From Table 2, it also shows that a 0.1 effort in keeping the environment clean/burying of placenta discharge after birth or abortion may result in a 2.70 reduction in bacterial load in the affected herds' environment and a corresponding reduction in the number of asymptomatic and symptomatic by 1.78, respectively. Interestingly, we notice that a 1% change in natural death may reduce the reproduction number by 0.95, but natural death cannot be used as a control measure; it, therefore, presupposes that the disease could be established as long as cattle live longer in an environment that is induced by the bacteria when there are no control measures; hence, vaccinating cattle and good environmental hygiene in farms will help mitigate the intensity of the organism on farms. It is well noticing that the analysis done so far represents the local sensitivity analysis of parameters in the model, which shows a close relationship between some of the parameters; hence, to see the global effect of these parameters on the model, we adopt the Latin hypercube sampling (LHS) and partial rank correlation coefficient (PRCC) which is demonstrated in the next Subsection 3.2.1.

#### 3.2.1. Global Sensitivity Analysis

In carrying out the global sensitivity analysis on the control reproduction number *ℛ*_*C*_, we use the analytic expression in equation (16) and the range of values in Table 1. The objective is to ascertain the most influential parameters in *ℛ*_*C*_ and also give some insightful ideas from the PRCC plot, by using a sample size of 1000.

The scatter plot in Figure 2(a) shows a positive relation between *ℛ*_*C*_ and the parameters *β*, Λ, *η*, *θ*, *α* and a negative relation between *ℛ*_*C*_ and the parameters *ν* and *ρ*. The positive relation indicates that a high rate of either of these parameters *β*, Λ, *η*, *θ*, *α* will produce a high transmission rate during an outbreak, while the negative relation for *ν* and *ρ* indicates that an increase in parameter value of *ν* and *ρ* will help reduce the severity of the disease-induced rate. Figure 2(b) gives sharply defined simulation results of the scatter plots in Figure 2(a) and that of the numerical signs in Table 2, but we notice a reverse sign for *α*, which is as a result of a decrease in the culling/isolation of symptomatic dairy cattle, as indicated in Remark 1; therefore, as shown in Figure 2(b) that, to have a positive impact of *c* in combination of other control measures so as to reduce *α*, *c* should be greater than 0.12, and thus it is represented by the red line as *c*_vital_. Figure 2(b) also indicates that the most influential parameters that promote the disease spread are *β*, *η*, and *α* (thus with low culling/isolation rate) and the most influential control parameter is *ε* (environmental hygiene). Therefore, any measures to reduce the relative relevance of *β*, *η*, and *α* (with an increase in culling/isolation rate) will reduce the spreading rate of the bacteria in dairy cattle herds.  Figure 2(c) shows the saturation effect of vaccination and environmental hygiene, and thus having *ℛ*_*C*_ < 1 and *ν*, *ε* > 0.5 may lead to a *Coxiella*-free environment.

## 4. Optimal Control Problem

Whenever the number of unknown parameters in a model is larger than the number of state equations, then the model in question usually allows for more than one solution. The optimization problem for such situations in continuous models is known as an optimal control problem. Here, we investigate the impact of time-variant controls: culling, vaccination, and environmental hygiene (cleaning/burying of placenta discharge after birth or abortion). This is to obtain the best control measure in preventing an outbreak of the disease in a situation where the cause of new infections is solely environmental. Thus, we seek to find optimal trajectories that show the relative importance of applying vaccination, culling, and environmental hygiene in controlling Q fever in an endemic setting. To see the impact of these controls, we modify model (1), thus setting *ρ*=0 (no natural resistance). The objective function is to reduce the numbers of asymptomatic cattle, symptomatic cattle, and bacterial load through vaccination *ν*, culling *c*, and environmental hygiene *ε*, respectively, and the cost associated with the implementation of the controls. Thus *u*_1_(*t*) ∈ *U*_ad_ is the percentage of susceptible and asymptomatic cattle who are vaccinated per unit time, *u*_2_(*t*) ∈ *U*_ad_ is the percentage of symptomatic cattle who are culled per unit time, and *u*_3_ ∈ *U*_ad_ is admissible measure for keeping the environment clean. Here *U*_ad_={*u*_*i*_|*u*_*i*_(*t*) are all  Lebesgue measurable and 0 ≤ *u*_*i*_(*t*) ≤ *u*_max_,  *t* ∈ [0, *t*_*f*_]}, where *i*=1,2,3 and *t*_*f*_ is the final time for running the control measures. Now the objective function that minimizes our desirable control problem is given as

(58) $$\begin{array}{c} J\left(u_{i}\left(t\right)\right)=\underset{u_{i},i=1,2,3}{\text{min}}\int_{0}^{t_{f}}\left[W_{1}A\left(t\right)+W_{2}I\left(t\right)+W_{3}B\left(t\right)+\frac{C_{1}}{2}u_{1}^{2}\left(t\right)+\frac{C_{2}}{2}u_{2}^{2}\left(t\right)+\frac{C_{3}}{2}u_{3}^{2}\left(t\right)\right]\text{d}t, \end{array}$$

subject to

(59) $$\begin{array}{c} \left\{\begin{array}{c} \frac{\text{d}S\left(t\right)}{\text{d}t}=\Lambda -\left[1-u_{3}\left(t\right)\right]\beta B\left(t\right)S\left(t\right)-\left(\mu +u_{1}\left(t\right)\right)S\left(t\right)+\theta V\left(t\right),\\ \frac{\text{d}V\left(t\right)}{\text{d}t}=u_{1}\left(t\right)S\left(t\right)-\left(\theta +\mu \right)V\left(t\right),\\ \frac{\text{d}A\left(t\right)}{\text{d}t}=\left[1-u_{3}\left(t\right)\right]\beta B\left(t\right)S\left(t\right)-\left(\mu +\alpha \right)A\left(t\right),\\ \frac{\text{d}I\left(t\right)}{\text{d}t}=\alpha A\left(t\right)-\left(u_{2}\left(t\right)+\mu \right)I\left(t\right),\\ \frac{\text{d}B\left(t\right)}{\text{d}t}=\eta \left(A\left(t\right)+I\left(t\right)\right)-\left(u_{3}\left(t\right)+\delta \right)B\left(t\right), \end{array}\right. \end{array}$$

with initial conditions

(60) $$\begin{array}{c} S\left(0\right)=S_{0}>0,\\ V\left(0\right)=V_{0}>0,\\ A\left(0\right)=A_{0}\geq 0,\\ I\left(0\right)=I_{0}\geq 0,\\ B\left(0\right)=B_{0}\geq 0, \end{array}$$

where [1 − *u*_3_(*t*)]*β* is the reduction in transmission due to environmental hygiene, *W*_*i*_,  *i*=1,2,3 are small positive constant to keep balance in the size of *S*(*t*), *A*(*t*), *I*(*t*), and *B*(*t*), respectively [31]. The square term of the controls reflects the nonlinearity in the cost of controls, and the half-term minimizes the effect of applying the controls [32]. The positive constants *C*_1_, *C*_2_, *C*_3_ which are associated with *u*_*i*_(*t*) are weight values such that 0 < *C*_1_,  *C*_2_ < *N*, and 0 < *C*_3_ < *B*. The relative weights given to the positive constants associated with the control terms indicate greater or lower importance placed on minimizing the cost of a control measure [33]. Now to obtain the optimal solutions, we defined a Lagrangian *L* for the control problem, given as follows:

(61) $$\begin{array}{c} L\left(S,A,I,B,u_{1},u_{2},u_{3}\right)=W_{1}A\left(t\right)+W_{2}I\left(t\right)+W_{3}B\left(t\right)+\frac{C_{1}}{2}u_{1}^{2}\left(t\right)+\frac{C_{2}}{2}u_{2}^{2}\left(t\right)+\frac{C_{3}}{2}u_{3}^{2}\left(t\right). \end{array}$$

We now seek the minimal value of the Lagrangian function. Hence, we obtain a Hamiltonian *H* for the control problem using Pontryagin's maximum principle:

(62) $$\begin{array}{c} H\left(S,A,I,B,u_{i},\lambda_{j},t\right)=L+\lambda_{1}\left(t\right)\frac{\text{d}S\left(t\right)}{\text{d}t}+\lambda_{2}\left(t\right)\frac{\text{d}V\left(t\right)}{\text{d}t}+\lambda_{3}\left(t\right)\frac{\text{d}A\left(t\right)}{\text{d}t}+\lambda_{4}\left(t\right)\frac{\text{d}I\left(t\right)}{\text{d}t}+\lambda_{5}\left(t\right)\frac{\text{d}B\left(t\right)}{\text{d}t}, \end{array}$$

where *λ*_*j*_,  *j*=1,2,3,4,5 are the adjoint variables associated with the states *S*, *V*, *A*, *I*, and *B*. Finally, taking the partial derivatives of *H* with respect to the state variables and the controls, we obtain the following relation for the adjoint and the optimal controls:

(63) $$\begin{array}{c} \frac{\text{d}\lambda_{1}}{\text{d}t}=-\frac{\partial H}{\partial S},\\ \frac{\text{d}\lambda_{2}}{\text{d}t}=-\frac{\partial H}{\partial V},\\ \frac{\text{d}\lambda_{3}}{\text{d}t}=-\frac{\partial H}{\partial A},\\ \frac{\text{d}\lambda_{4}}{\text{d}t}=-\frac{\partial H}{\partial I},\\ \frac{\text{d}\lambda_{5}}{\text{d}t}=-\frac{\partial H}{\partial B},\\ u_{1}^{*}=\frac{\partial H}{\partial u_{1}}=0,\\ u_{2}^{*}=\frac{\partial H}{\partial u_{2}}=0,\\ u_{3}^{*}=\frac{\partial H}{\partial u_{3}}=0, \end{array}$$

with transversality conditions (or boundary conditions) [31], *λ*_*j*_(*t*_*f*_)=0,  *j*=1,2,3,4,5. Therefore, the adjoint system is given as

(64) $$\begin{array}{c} \frac{\text{d}\lambda_{1}}{\text{d}t}=\lambda_{1}\left(\mu +u_{1}-B\beta \left(u_{3}-1\right)\right)-\lambda_{2}u_{1}+B\beta \lambda_{3}\left(u_{3}-1\right),\\ \frac{\text{d}\lambda_{2}}{\text{d}t}=\lambda_{2}\left(\mu +\theta \right)-\lambda_{1}\theta ,\\ \frac{\text{d}\lambda_{3}}{\text{d}t}=\lambda_{3}\left(\alpha +\mu \right)-\alpha \lambda_{4}-\eta \lambda_{5}-W_{1},\\ \frac{\text{d}\lambda_{4}}{\text{d}t}=\lambda_{4}\left(\mu +u_{2}\right)-W_{2}-\eta \lambda_{5},\\ \frac{\text{d}\lambda_{5}}{\text{d}t}=\lambda_{5}\left(\delta +u_{3}\right)-W_{3}-S\beta \lambda_{1}\left(u_{3}-1\right)+S\beta \lambda_{3}\left(u_{3}-1\right). \end{array}$$

By the optimality conditions, we have

(65) $$\begin{array}{c} \frac{\partial H}{\partial u_{1}}≔C_{1}u_{1}-S\lambda_{1}+S\lambda_{2}=0,\\ \frac{\partial H}{\partial u_{2}}≔C_{2}u_{2}-I\lambda_{4}=0,\\ \frac{\partial H}{\partial u_{3}}≔C_{3}u_{3}-B\lambda_{5}+BS\beta \lambda_{1}-BS\beta \lambda_{3}=0,\\ ⟹u_{1}^{*}=\frac{S\lambda_{1}-S\lambda_{2}}{C_{1}},\\ u_{2}^{*}=\frac{I\lambda_{4}}{C_{2}},\\ u_{3}^{*}=\frac{B\lambda_{5}-BS\beta \lambda_{1}+BS\beta \lambda_{3}}{C_{3}}. \end{array}$$

Using the optimal solution in the control space [31], we have

(66) $$\begin{array}{c} u_{1}^{*}=\left\{\begin{array}{cc} 0, & \text{if }\frac{S^{*}\lambda_{1}-S^{*}\lambda_{2}}{C_{1}}\leq 0,\\ \frac{S^{*}\lambda_{1}-S^{*}\lambda_{2}}{C_{1}}, & \text{if }0<\frac{S^{*}\lambda_{1}-S^{*}\lambda_{2}}{C_{1}}<0.9,\\ 0.9, & \text{if }\frac{S^{*}\lambda_{1}-S^{*}\lambda_{2}}{C_{1}}\geq 0.9, \end{array}\right.\\ u_{2}^{*}=\left\{\begin{array}{cc} 0, & \text{if }\frac{\lambda_{4}I^{*}}{C_{2}}\leq 0,\\ \frac{\lambda_{4}I^{*}}{C_{2}}, & \text{if }0<\frac{\lambda_{4}I^{*}}{C_{2}}<1,\\ 1, & \text{if }\frac{\lambda_{4}I^{*}}{C_{2}}\geq 1, \end{array}\right.\\ u_{3}^{*}=\left\{\begin{array}{cc} 0, & \text{if }\frac{B^{*}\lambda_{5}-B^{*}S^{*}\beta \lambda_{1}+B^{*}S^{*}\beta \lambda_{3}}{C_{3}}\leq 0,\\ \frac{B^{*}\lambda_{5}-B^{*}S^{*}\beta \lambda_{1}+B^{*}S^{*}\beta \lambda_{3}}{C_{3}}, & \text{if }0<\frac{B^{*}\lambda_{5}-B^{*}S^{*}\beta \lambda_{1}+B^{*}S^{*}\beta \lambda_{3}}{C_{3}}<0.8,\\ 0.8, & \text{if }\frac{B^{*}\lambda_{5}-B^{*}S^{*}\beta \lambda_{1}+B^{*}S^{*}\beta \lambda_{3}}{C_{3}}\geq 0.8. \end{array}\right. \end{array}$$

Therefore, the compact notation (characterization) of the admissible control is then written as

(67) $$\begin{array}{c} u_{1}^{*}=\text{min}\left\{\text{max}\left(0,\frac{\lambda_{1}\left(S^{*}-B^{*}S^{*}\beta \right)-S^{*}\lambda_{2}+B^{*}S^{*}\beta \lambda_{3}}{C_{1}}\right),0.9\right\},\\ u_{2}^{*}=\text{min}\left\{\text{max}\left(0,\frac{\lambda_{4}I^{*}}{C_{2}}\right),1\right\},\\ u_{3}^{*}=\text{min}\left\{\text{max}\left(0,\frac{\lambda_{5}B^{*}-\lambda_{1}\beta B^{*}S^{*}+\lambda_{3}\beta B^{*}S^{*}}{C_{3}}\right),0.8\right\}. \end{array}$$

The control terms in the bound of 0.9 and 0.8 satisfy the inability to vaccinate all susceptible cattle at a time and a 0.2 failure in eliminating all bacteria in a particular farm at a given time, note that the control bounds are assumed for a realistic illustrative purposes and may not be practically practised, but the variation of these bounds will not narrow the essence of the studies. Substituting (67) into (59), (62), and (64), respectively, we obtain an optimality system with the corresponding Hamiltonian system *H*^*∗*^ which is then solved numerically to obtain optimal trajectories for our dynamical system.

### 4.1. Optimal Control Trajectories

**Figure 3:**
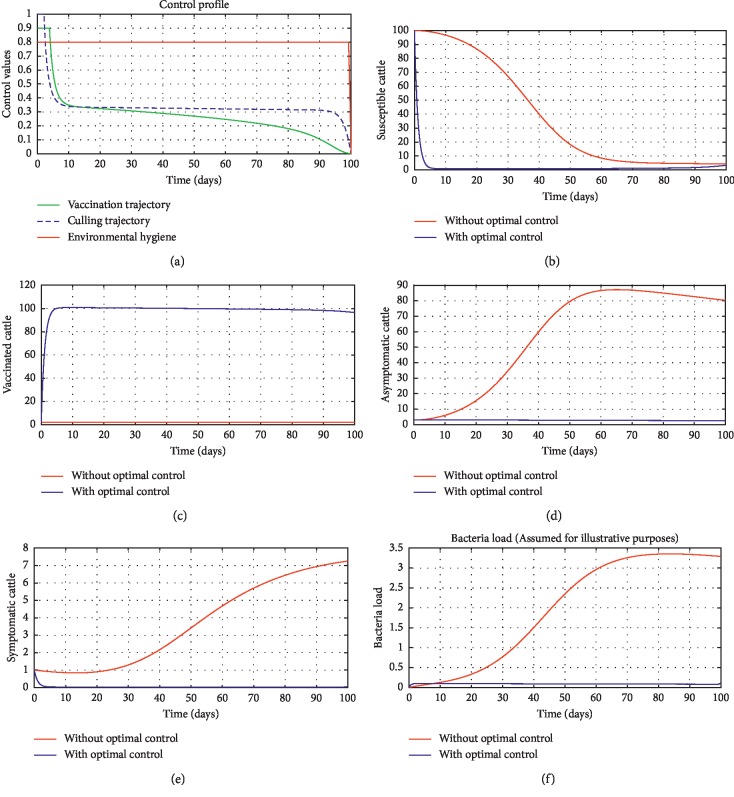
Optimal trajectory results with and without controls. All initial state values are assumed for illustrative purposes. (a) Control profile. (b) Susceptible cattle. (c) Vaccinated cattle. (d) Asymptomatic cattle. (e) Symptomatic cattle. (f) Bacterial load.

**Figure 4:**
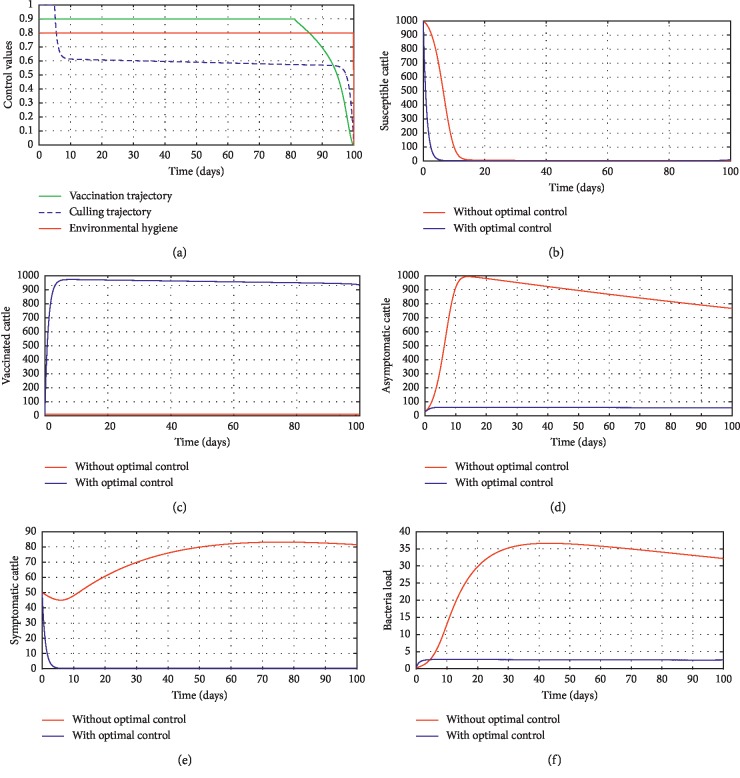
Optimal trajectory results with and without controls, with a change in initial state value data. All initial state values are assumed for illustrative purposes. (a) Control profile. (b) Susceptible cattle. (c) Vaccinated cattle. (d) Asymptomatic cattle. (e) Symptomatic cattle. (f) Bacterial load.

In order to obtain the optimal trajectories for model (59), we employ the forward-backward sweep method, as described in [34]. We begin with an initial guess for the controls. The state equation (59) is solved forward in time while the adjoint function (64) is solved backward in time. The controls are then updated for a period of time until convergence is achieved. For better numerical output, we firstly made use of the following initial state values: *S*_0_=100,  *V*_0_=2,  *A*_0_=3,  *I*_0_=1,  *B*(0)=0.0025 and the stated parameter values in Table 1. The initial bacterial load is assumed to be small, the reason being that per the numerical simulations, the bacterial population grows more and more rapidly as depicted in Figures 3(f) and 4(f), respectively, by the red line, hence, the need to start with small initial values for the bacterial load so as to get a suitable numerical output for that of the initial conditions chosen for the animal population. The corresponding weight in the objective function (58) is taken to be *W*_1_=2,  *W*_2_=4,  *W*_3_=2,  *C*_1_=1,  *C*_2_=1,  *C*_3_=0.2. The figures below depict the relative importance of finding optimal strategy/strategies in controlling the disease spread in cattle (animal population).  Table 3 shows the total cost associated with the objective function (58) after a simulation time of 100 days. Secondly, we look at the control trajectories and the accompanying cost by changing the associated weights of control, cost of control implantation, and initial state values and thus *W*_1_=1,  *W*_2_=1,  *W*_3_=2,  *C*_1_=1,  *C*_2_=1,  *C*_3_=0.5 and *S*_0_=1000,  *V*_0_=10,  *A*_0_=30,  *I*_0_=50, *B*(0)=0.25 as depicted in Figure 4 and Table 3, item 3.

Figure 3(a) shows the control profile for the various bounds employed in model (59), which indicates that a bound of 0.8 for environmental hygiene is maintained for the entire simulation time, but a drop in the control bound for both vaccination and culling. Thus, the bound for culling and vaccination switches to 0.32 after 10 days. This indicates during the initial onset of an outbreak, effort should be made to cull all symptomatic cattle or isolate them from other susceptible (healthy) cattle for the first 10 days, after which less culling approach can be adopted, and also for first 10 days, at least 90% of cattle should receive vaccination during the initial stage of the disease outbreak. Figures 3(b) and 3(c) show that applying vaccination, culling and environmental hygiene simultaneously increases the rate of healthy cattle, thereby reducing the risk of infection in the susceptible cattle population and also reducing the number of asymptomatic cattle, symptomatic cattle, and bacterial load in the environment as shown in Figures 3(d)–3(f), respectively. We also notice that the gradual use of these controls in the long run is more cost-effective as shown in Table 3, item 2.

In Figure 4(a), we changed the initial population size, which indicates that an increase in population size requires that the initial control bound for vaccination be kept for 80 days, and thus an increase in population size of dairy cattle herds requires high control effort and high resources as depicted in item 3 of Table 3. The control trajectory for the vaccination bound and Figure 4(c) further indicate that for optimal eradication or reduction of the disease in a population of 1000 cattle (livestock), at least 80% of the animals should be vaccinated within the first 20–80 days. We also noticed that the use of vaccination, culling, and environmental hygiene has similar control efficacy in the disease compartments irrespective of the population size as shown in Figures 4(b)–4(f), respectively.

**Figure 5:**
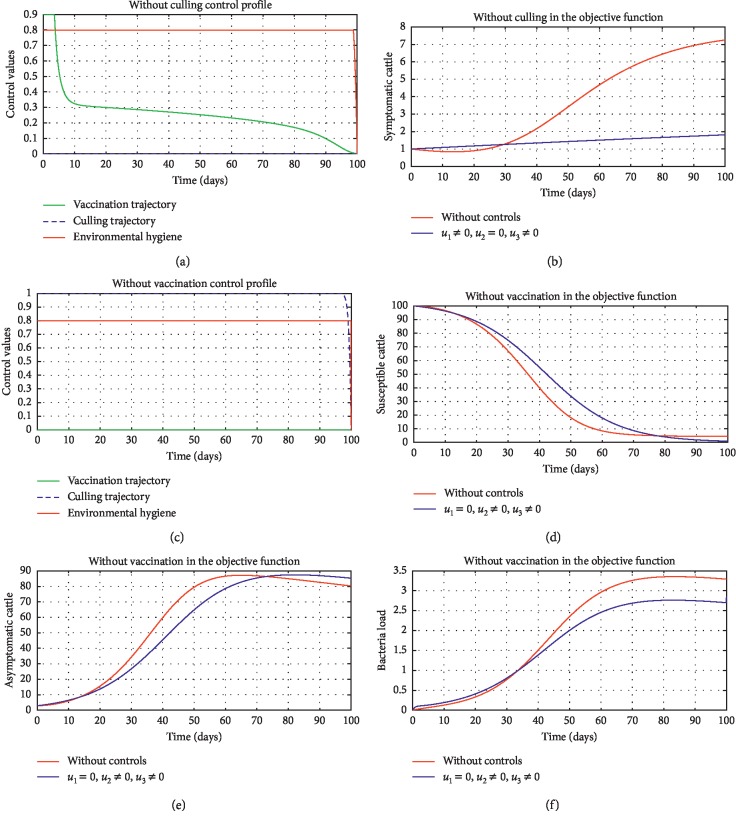
Optimal trajectory results marked by diversity in control procedures. All initial state values are assumed for illustrative purposes. (a) Without culling control profile. (b) Without culling in the objective function. (c) Without vaccinated control profile. (d) Without vaccination in the objective function. (e) Asymptomatic cattle (without vaccination in the objective function). (f) Bacterial load (without vaccination in the objective function).

**Figure 6:**
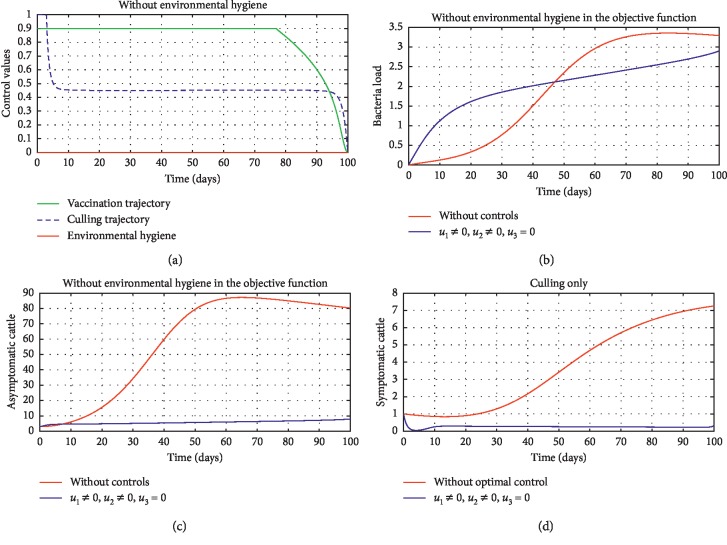
Optimal trajectory results marked by diversity in control procedures. All initial state values are assumed for illustrative purposes. (a) Control values (no environmental hygiene). (b) Bacterial load (no environmental hygiene). (c) Asymptomatic cattle (no environmental hygiene). (d) Symptomatic cattle (culling only).

In Figure 5, we considered variations that may arise in the control measures, thus sticking to one or more controls. In Figure 5(a), we assumed that *u*_2_=0, that is a situation where culling is not applied, and it reveals that symptomatic cattle will always be in the population but stays below the trajectory when all controls are zero (see Figure 5(b)). Therefore, we notice that in a situation where a farm owner chooses to ignore culling and decide to stick to only vaccination and environmental hygiene during an outbreak of the disease may not lead to the eradication of the disease; hence, the combination of vaccination and environmental hygiene is not a good control measure in reducing the number of symptomatic cattle (see Figure 6(d)) for the implementation of culling only as a control measure on the symptomatic compartment.  Figure 5(c) shows that in the absence of vaccination, the control bound for culling should remain at 1, which means that during the cause of culling, the aim should be focused on destroying all infected farms or animals which is sometimes practically hard to achieve and also comes with high control cost as shown in Table 3, item 7. Therefore, it clearly shows in Figures 5(d)–5(f) that effective vaccination plays a major role in reducing the disease-induced rate in dairy cattle herds depending only on culling. It can also be seen that without vaccination, the trajectories of Figures 5(d)–5(f) of the optimal control stay very close to the trajectories without optimal control.

Figure 6(a) shows the control profile in the absence of environmental hygiene; the figure depicts that with only vaccination and culling as control measures, the bound of vaccination is kept at 0.9 for 78 days before switching to 0.8, and the bound of culling switches to 0.45 as compared to Figure 3(a). Hence, Figure 6(b) depicts the noneffectiveness of vaccination and culling on the bacterial load in the environment until day 47, but due to vaccination, the number of asymptomatic cattle reduces as shown in Figure 6(c). Figure 6(d) shows the effect of culling on the number of symptomatic cattle, which indicates that early detection of symptomatic cattle on the farm will help in mitigating the disease within the first 5 days.

## 5. Concluding Remarks

We presented a theoretical analysis of Q fever within dairy cattle herds, which may lead to new research on the disease dynamics, such as the effect of direct transmission, seasonal transmission, the dynamical role of ticks in maintaining the circle of Q fever in livestock, the role of media in averting the disease in humans, and the dynamical spread of the disease between livestock and humans. Here we analytically obtained the control states and numerically simulated it for different outcomes. We notice that culling only as a control measure produces a higher control cost, which also does not completely eradicate the number of symptomatic cattle due to the asymptomatic nature of the disease. We obtained a cost profile for the various control measures as shown in Table 3 which suggests that the most effective method in getting a lower control cost, in the long run, is the continuous application of vaccination and good environmental hygiene together with culling or isolation of symptomatic cattle. The numerical simulation also reveals that irrespective of the control employed, the disease will always be present in the animal population, but vaccinating and cleaning/burying of placenta discharge after birth or abortion will help to mitigate the disease in the animal population. In totality, our work has revealed some global dynamics of Q fever in dairy cattle herds and the impact of sensitivity analysis and optimal control strategies in managing Q fever disease in dairy cattle herds (livestock). We also analytically obtained the local and global stability analysis of the disease by using matrix-theoretic method and a Lyapunov functional. The model studied here did not assume a complex model, but the results can provide some ideas for further studies and the prevention of the disease.

## Figures and Tables

**Table 1:** Model parameter descriptions and values.

Parameter	Description	Range	Baseline value	Reference
*β*	Environmental transmission rate (indirect)	0.0030–0.0943	0.0943 day^−1^/cattle	[19]
Λ	Birth/recruitment rate	0.0034–0.0036	0.0036 day^−1^	Estimated
*η*	Proportion of bacteria shed through mucus/faeces filling the environment	0.0021–0.0400	0.0400 day^−1^/cattle	[19, 20]
*ρ*	Transition rate from *A* to *S*	0.0774–0.1208	0.1000 day^−1^	[19, 20]
*α*	Transition rate *A* to *I*	0.0001–0.0117	0.0029 day^−1^	[19, 20]
*θ*	Loss of vaccine immunity	0.0027–2.0000	0.0027 day^−1^	[20]
*μ*	Natural death rate of cattle	0.0001–0.0002	0.0002 day^−1^	Estimated
*ν*	Vaccination rate	0.1000–0.9000 unitless	0.1000	Assumed
*ε*	Environmental hygiene	0.1000–0.9000 unitless	0.1000	Assumed
Δ	Natural decay of bacteria from the dust/soil	0.0067–0.0083	0.0083 day^−1^	Estimated
*c*	Culling/mortality rate	0.0008–0.0286	0.0286 day^−1^	[20]

**Table 2:** The elasticity indices of *ℛ*_*C*_=1.5193 and the relative change of *A*^*∗*^, *I*^*∗*^, and *B*^*∗*^ at the endemic equilibrium points, to the parameters in the model.

Parameter	Baseline value	Elasticity index, *ℛ*_*C*_	*A* ^*∗*^	*I* ^*∗*^	*B* ^*∗*^
*β*	0.0943	+1.0000	+1.9255	+1.9255	+1.9255
Λ	0.0036	+1.0000	+2.9255	+2.9255	+2.9255
*η*	20.0400	+1.0000	+1.9255	+1.9255	+2.9255
*ρ*	20.0097	−0.7578	−1.4592	−1.4592	−1.4592
*α*	10.0029	−0.1351	−1.1956	−0.1956	−1.1041
*θ*	10.0027	+0.9048	+1.7422	+1.7422	+1.7422
*μ*	10.0002	−0.9492	−1.8923	−1.8992	−1.8929
*ν*	10.1000	−0.9718	−1.8713	−1.8713	−1.8713
*ε*	10.1000	−0.9234	−1.7779	−1.7779	−2.7013
*δ*	20.0083	−0.0766	−0.1476	−0.1476	−0.2242
*c*	0.0286	−0.0908	−0.1749	−1.1680	−0.2658

**Table 3:** The total cost *J* in regard to different control strategies associated with model (1) with *ρ*=0.

Item	Strategies	Associated cost *J*
1	Without optimal control, *u*_1_=0, *u*_2_=0, *u*_3_=0	28,977.63
2	With optimal control, *u*_1_ ≠ 0, *u*_2_ ≠ 0, *u*_3_ ≠ 0	623.04
3	With optimal control, *u*_1_ ≠ 0, *u*_2_ ≠ 0, *u*_3_ ≠ 0 with *S*_0_=1000	6,537.69
4	Culling only, *u*_1_=0, *u*_2_ ≠ 0, *u*_3_=0	23,057.75
5	Without environmental hygiene, *u*_1_ ≠ 0, *u*_2_ ≠ 0, *u*_3_=0	1,616.53
6	Without culling, *u*_1_ ≠ 0, *u*_2_=0, *u*_3_ ≠ 0	1,191.37
7	Without vaccination, *u*_1_=0, *u*_2_ ≠ 0, *u*_3_ ≠ 0	10,722.43

## Data Availability

The parameter values (data) used to support the findings of this study have been described in Section 3, and other data (parameter values) used were previously reported data and are cited at relevant places within the text as references [19, 20].
